# Students’ perceptions of STEM learning after participating in a summer informal learning experience

**DOI:** 10.1186/s40594-018-0133-4

**Published:** 2018-09-21

**Authors:** Thomas Roberts, Christa Jackson, Margaret J. Mohr-Schroeder, Sarah B. Bush, Cathrine Maiorca, Maureen Cavalcanti, D. Craig Schroeder, Ashley Delaney, Lydia Putnam, Chaise Cremeans

**Affiliations:** 1 Bowling Green State University, 529 Education Building, Bowling Green, OH 43403 USA; 2 Iowa State University, School of Education, 2642A Lagomarcino Hall, 901 Stange Road, Ames, IA 50011 USA; 3 University of Kentucky, 105 Taylor Education Building, Lexington, KY 40506-0001 USA; 4 University of Central Florida, School of Teaching, Learning, and Leadership, College of Education and Human Performance, P.O. Box 161250, Orlando, FL 32816-1250 USA; 5 Department of Teacher Education, California State University, Long Beach, 1250 Bellflower Blvd, Long Beach, CA 90840 USA; 6 The Ohio State University, College of Medicine, 370 W 9th Avenue, Columbus, OH 43210 USA; 7 Fayette County Public Schools, 1224 Kannapolis Place, Lexington, KY 40513 USA; 8 Morehead State University, 4156 Starrush Place, Lexington, KY 40509 USA

**Keywords:** STEM education, Informal learning, Student learning, Student perceptions

## Abstract

**Background:**

Informal learning environments increase students’ interest in STEM (e.g., Mohr‐Schroeder et al. School Sci Math 114: 291–301, 2014) and increase the chances a student will pursue a STEM career (Kitchen et al. Sci Educ 102: 529–547, 2018). The purpose of this study was to examine the impact of an informal STEM summer learning experience on student participants, to gain in-depth perspectives about how they felt this experience prepared them for their in-school mathematics and science classes as well as how it influenced their perception of STEM learning. Students’ attitudes and perceptions toward STEM are affected by their motivation, experience, and self-efficacy (Brown et al. J STEM Educ Innov Res 17: 27, 2016). The academic and social experiences students’ have are also important. Traditionally, formal learning is taught in a solitary form (Martin Science Education 88: S71–S82, 2004), while, informal learning is brimming with chances to connect and intermingle with peers (Denson et al. J STEM Educ: Innovations and Research 16: 11, 2015).

**Results:**

We used a naturalistic inquiry, phenomenological approach to examine students’ perceptions of STEM while participating in a summer informal learning experience. Data came from students at the summer informal STEM learning experiences at three diverse institutions across the USA. Data were collected from reflection forms and interviews which were designed to explore students’ “lived experiences” (Van Manen 1990, p. 9) and how those experiences influenced their STEM learning. As we used a situative lens to examine the research question of how participation in an informal learning environment influences students’ perceptions of STEM learning, three prominent themes emerged from the data. The informal learning environment (a) provided context and purpose to formal learning, (b) provided students opportunity and access, and (c) extended STEM content learning and student engagement.

**Conclusions:**

By using authentic STEM workplaces, the STEM summer learning experience fostered a learning environment that extended and deepened STEM content learning while providing opportunity and access to content, settings, and materials that most middle level students otherwise would not have access to. Students also acknowledged the access they received to hands-on activities in authentic STEM settings and the opportunities they received to interact with STEM professionals were important components of the summer informal learning experience.

## Background

In the United States, we currently face a shortage of science, technology, engineering, and mathematics (STEM) majors and graduates (National Science Board 2016; The Committee on STEM Education National Science and Technology Council 2013) while at the same time STEM occupations are expected to grow (Langdon et al. 2011; U.S. Bureau of Labor Statistics 2018). This two-fold issue necessitates STEM education in the U.S. becomes and remains a priority. According to the National Research Council (2011), this priority must include broadening women’s and minorities’ participation in STEM and increasing STEM literacy for all students, regardless of whether they plan to pursue a STEM major or career. Informal learning environments have been shown to increase students’ interest in STEM (e.g., Mohr-Schroeder et al. 2014) and have been shown to increase the chances a student will pursue a STEM career (Kitchen et al. 2018; Kong et al. 2014).

Researchers identify interest and motivation as important components in inspiring students to pursue STEM learning because it contributes to students’ learning and success in retaining STEM content (Bell et al. 2009). In response to President Barack Obama’s Call to Action (The President’s Council of Advisors on Science and Technology (PCAST 2010), states, school districts, and individual schools, as well as researchers in the United States (U.S.) have increased their focus on improving students’ motivation and interest in STEM, particularly at the middle school level. According to Brown et al. (2016), the educational deficit in STEM areas has led to a workforce gap in many STEM professions. Informal learning environments can support student STEM knowledge and skills (e.g., Denson et al. 2015), positively impact student interest in STEM (e.g., Denson et al. 2015; Mohr-Schroeder et al. 2014), and increase the likelihood to pursue a STEM career while attending college (Kitchen et al. 2018; Kong et al. 2014).

### Targeting elementary and middle school students for STEM

Studies have shown that students who have an increased interest in science, mathematics, and engineering in the early years of their education are more likely to pursue that interest resulting in a STEM-related career (After-School Alliance 2015). Unfortunately, before the eighth grade, many students have concluded that the STEM subjects are too challenging, boring, and/or uninteresting (PCAST 2010), which limits their participation in STEM subjects and activities. Research has shown the importance of motivating students to learn STEM content in the elementary and middle grades. “Students who express interest in STEM in eighth grade are up to three times more likely to ultimately pursue STEM degrees later in life than students who do not express such an interest” (PCAST 2010, p. 19). Research has shown that students’ learning is delayed during summer breaks (McCombs et al. 2011), and students from low-socioeconomic households have more knowledge loss during summer months due to their lack of access to summer learning. Furthermore, summer learning deficits are accumulated and by ninth grade, two-thirds of the receivement gap (Chambers 2009) among low socioeconomic students can be attributed to unequal access to summer learning experiences (Alexander et al. 2007; McCombs et al. 2011). Therefore, it is imperative to *prepare* and *inspire* each and every student, specifically students of color, females, and students from low socioeconomic backgrounds, to learn STEM (PCAST 2010).

### Informal learning experiences

Informal STEM learning experiences have the potential to support students’ learning and engagement in a formal STEM learning environment. Informal STEM learning experiences address the limitations of the formal school experience by providing opportunities (Bell et al. 2009; Meyers et al. 2013, Popovic and Lederman 2015) that build students’ awareness of and interest in the STEM fields. Students who struggle in the formal and more traditional STEM courses tend to be more interested and motivated in STEM when it is presented in a more engaging, hands-on way. Informal STEM learning environments are naturally composed in a way to promote learning through real-world modeling and examples (Martin 2004; Meredith 2010; Popovic and Lederman 2015). Informal STEM learning environments also help students understand concepts and their ability to recall information (Allsopp et al. 2007; Popovic and Lederman 2015). Participation in short-term STEM summer experiences (Bell et al. 2009; Kitchen et al. 2018; King 2017; Mohr-Schroeder et al. 2014) or other long-term informal STEM programs (After-School Alliance 2011; Baran et al. 2016; Barker et al. 2014; Klanderman et al. 2013; Massey and Lewis 2011) have been shown to increase students’ interest in STEM.

### Factors that influence students’ perceptions of STEM

Students’ attitudes and perceptions toward STEM are affected by their motivation, experience, and self-efficacy (Brown et al. 2016; Turner and Patrick 2004; Weinberg et al. 2011). Brown et al. (2016) studied the relationships between STEM curriculum and students’ attitudes and found student interest played a more important role in intention to persist in STEM when compared with self-efficacy. These discrepancies may be remedied by exposing students to a greater longevity of experience with activities which foster self-determination and interest-led, inquiry-based projects (Boekaerts 1997; Honey et al. 2014; Moote et al. 2013).

The academic and social experiences students’ have are also important. More specifically, middle level students are especially impacted by peers because:

During adolescence, students are often reluctant to do anything that causes them to stand out from the group, and many middle-grades students are self-conscious and hesitant to expose their thinking to others. Peer pressure is powerful, and a desire to fit in is paramount. (NCTM, 2000, p. 268)

Traditionally, formal learning is taught in a solitary form (Martin 2004), while informal learning is brimming with chances to connect and intermingle with peers (Barker et al. 2014; Denson et al. 2015; Klanderman et al. 2013).

Many educators approach work with students through a problem-solving framework to develop positive STEM perceptions. The STEM Task Force Report (2014) argued for the use of problem-solving and project-based frameworks because of their use of “real-world issues [which] can enhance motivation for learning and improve student interest, achievement, and persistence” (p. 9). Important to the positive STEM perception development of underrepresented students in STEM are opportunities to participate in authentic STEM learning experiences. For these reasons, a need exists for informal learning environments, such as the See Blue See STEM model*,* to provide students with meaningful exposure to a STEM community in which to participate, practice, and belong (O'Connell et al. 2017).

Our work directly aligns to the priorities outlined by the National Research Council as we provide informal STEM learning opportunities to elementary and middle school students—focusing on students from underrepresented populations in STEM (i.e., Black, Latinx, Native American, and females). We believe in STEM literacy for each and every student is feasible and can be supported by access and opportunities to authentic learning experiences. The purpose of this study is to examine the impact of an informal STEM summer learning experience on student participants, to gain in-depth perspectives about how students perceived this experience prepared them for their in-school mathematics and science classes as well as how it influenced their perception of STEM learning. The research question for this study was: How does participating in an informal learning environment influence middle level students’ perceptions of STEM learning?

### Theoretical framework

To examine how participation in an informal learning environment influences students’ perceptions of STEM learning, we used situated learning theory. Situated learning and related theoretical perspectives (i.e., cognitive apprenticeship) have been utilized in investigating the connections between informal learning and STEM education (e.g., Larkins et al. 2013). More generally, situated learning has been used to study learning and attitudes toward STEM (e.g., Guzey et al. 2016). Applied to perceptions of STEM learning, such a theoretical lens allows the experiences of students to be explored in the context of the authentic activity where students experience STEM learning.

Central to the situated perspective is how interactions between learner and environment (Brown et al. 1989; Kirshner and Whitson 1997; Lave 1991; Lave and Wenger 1991), mediated by social interactions create opportunities for learners to acquire knowledge. The learning that occurs arises from *legitimate peripheral participation* (Lave 1991; Lave and Wenger 1991) in authentic activity (Brown et al. 1989). Through opportunities to acquire and apply knowledge and practice skills, learners develop deeper understandings (Brown et al. 1989) from the meaningful context in which those opportunities exist (Luehmann 2009; Sullivan 2008). The community is an essential element of the meaningful context and is a powerful vehicle for transforming perspectives and understandings (e.g., Johri and Olds 2011). Informal learning promotes access and opportunities to participate in authentic STEM learning, and therefore, influences perceptions (NRC 2009).

Situated STEM learning results from an integration of STEM content within a community practice where “learning is authentic and relevant, therefore representative of an experience found in actual STEM practice” (Kelley and Knowles 2016, p. 4). The See Blue See STEM model is one such informal environment, with targeted efforts to reach student populations underrepresented in STEM and capitalize on the transformational potential of engaging students in hands-on interactive sessions with STEM professionals. The STEM teaching and learning summer experience mirrors that of the work of professionals in the field. Employing a situative perspective in this study provides a context for broadening understanding of how authentic experiences in an informal environment can transform students’ perceptions toward STEM learning across contexts.

## Methods

We used a naturalistic inquiry, phenomenological approach to examine students’ perceptions of STEM while participating in a summer informal learning experience. Naturalistic inquiry, falling in the constructivist paradigm, allows for multiple realities to be created by the students (Lincoln and Guba 1985). The meanings students create are constructed by their participation in specific settings (Crotty 1998). The phenomenological approach allowed us to focus on the “lived experience” of student participants in an informal learning environment (Creswell 2014). Students’ participation in the informal learning environment allows for the meaning students place on their experiences to be investigated (Merriam 2009).

### See blue see STEM summer informal learning experience model

The See Blue See STEM Summer Experience is a 1-week summer informal STEM learning experience for middle level students. Founded in 2010 in Kentucky, the See Blue See STEM model provides a variety of STEM content experiences for students to participate during the summer to spark their interest in STEM. The See Blue See STEM model’s goal is to expose middle level students, particularly underrepresented populations, to a variety of STEM fields and STEM professionals in their workplace environment through authentic, hands-on instruction to increase students’ interest in a STEM career. The See Blue See STEM model was named a Top 5 model for Broadening Participation at the 2015 EPSCoR National Conference (Mohr-Schroeder 2015), and was replicated at Iowa State University and California State University—Long Beach during Summer 2017.

Throughout the See Blue See STEM model, focus is given to ensure high-quality, authentic, hands-on sessions with STEM content faculty from Colleges of Engineering, Education, Arts and Sciences, Medicine, as well as STEM professionals and/or informal STEM learning partners. The selection of presenters, which varies from year-to-year, provides opportunities for students to engage in a variety of STEM fields in their authentic research environments. The eight mathematical practices (NGA Center for Best Practices and CCSSO 2010) and the eight science and engineering practices (NGSS Lead States 2013) are present throughout the sessions.

In the See Blue See STEM model, all students participate in robotics (e.g., Lego Mindstorm EV3, Vex) or EDISON, which provides an engaging and motivating platform for students to actively build, explore, investigate, inquire, and communicate together to develop their programming and problem-solving skills. Curriculum topics and content are different each year in order to allow repeating students to have exposure to a variety of STEM content. In addition, the students engage in different content sessions each day. Students engage in robotics or EDISON every day for 3 hours and content sessions the other 3 hours of the day (see Table 1 for a sample 2-day schedule). This model, similar to Kelley and Knowles’ (2016) approach to STEM, allows students to work in a community of practice that is situated in authentic contexts and facilitated by a STEM expert.

**Table 1:** Sample daily schedule used in See Blue See STEM Model

	Day 1	Day 2
9:00–9:30	Welcome, Introductions, Community Building	
9:30–10:00	Explorations in Biomedical Science: DNA Extraction	Modeling with 3D pens
10:00–10:30		
10:30–11:00		
11:00–11:30		
11:30–12:00		
12:00–12:30	Transition to Lunch, Lunch, Transition to Robotics	Transition to Lunch, Lunch, Transition to Robotics
12:30–1:00		
1:00–1:30	Robotics	Robotics
1:30–2:00		
2:00–2:30		
2:30–3:00		
3:00–3:30		
3:30–4:00		
4:00	Pick-up	

For example, California State University-Long Beach students completed the *Follow the Flow Challenge* with local engineers from a community partner organization. The local engineers from a community partner introduced engineering and described the career paths and college courses they took to become engineers. Then they introduced the challenge, *Follow the Flow*, where students designed and built “a water flow system to move beads through terraced layers” (Finio 2018). The engineers engaged with the students and supported them as they completed the engineering design process. As students designed, built, and tested their structures, the engineers fostered their thinking and allowed them to engage in both the Standards for Mathematical Practice (e.g., attend to precision) and NGSS Science and Engineering Practices (e.g., planning and carrying out investigations).

At the University of Kentucky, students modeled with 3-D pens. This session was facilitated by a professor of mathematics education, and the students used 3-D pens to create a variety of three-dimensional mathematical shapes (e.g., cube, dodecahedron, pyramid). Once students created and named the shapes, they were challenged to design structures that incorporated those shapes. The students designed, built, and improved their structures while attending to the mathematically important shapes they were using. This allowed them to engage in the engineering design process while also utilizing the SMPs (e.g., modeling with mathematics and using appropriate tools strategically) and the NGSS Science and Engineering Practices (such as planning and carrying out investigations and obtaining, evaluating, and communicating information) in a community of practice.

These examples illustrate the pedagogical approach used within the See Blue See STEM model. The students engage in authentic activities that are facilitated by experts in the field. Students work in a community of practice to plan, create, and refine their ideas by using the engineering design process. Technological tools are used when appropriate, such as the 3-D pens to create structures. Mathematics and science content knowledge is applied, while the emphasis is placed on the practices of these domains. In other words, students are engaging in the processes that are important components of the disciplines.

### Participants

In order to recruit students to attend the summer informal STEM learning experience, an informational flyer and website address is sent out via statewide listservs and to middle schools in the region where the summer experience is held. Although the summer experience is open to all students, the camp focuses on attracting underrepresented populations in STEM fields, especially females and students of color. We define underrepresented populations in STEM fields as female, Black, Hispanic/Latinx, American Indians or Alaska Natives, and Native Hawaiians or Other Pacific Islanders (National Science Foundation 2017). The directors of the summer learning experiences work directly with family resource directors at each of the area high needs elementary and middle schools in order to identify and specially invite underrepresented students. These students are guaranteed a place in the camp, provided a scholarship based on financial need, and provided transportation, if needed, to and from camp.

The summer informal STEM learning experience was comprised of incoming 5th–8th graders at all three sites. According to students’ self-identified data, the one institution’s summer experience population between 2012 and 2017 was 39% females, 8% Black, 5% Asian, 1% Hispanic/Latinx, 75% White, 5% other (e.g., mixed race), 6% no response, and 43% from underrepresented populations in STEM. The second institution’s summer experience population in 2017 was 55% females, 36% Black, 6% Asian, 39% Hispanic/Latinx, 15% White, and 3% other (e.g., mixed race), and 91% from underrepresented populations in STEM. The summer experience population in 2017 at the third institution was 59% females, 76% Hispanic/Latinx, 12% Asian, 12% other (e.g., mixed race), and 94% from underrepresented populations in STEM.

### Data collection

Data for this paper came from students at the summer informal STEM learning experiences at the three diverse institutions across the United States. Data were collected from reflection forms and interviews which were designed to explore students’ “lived experiences” (Van Manen 1990, p. 9) and how those experiences influenced their STEM learning. During the last 2 days of the week of the summer informal learning experience, student participants, for which we had IRB consent and assent, participated in a semi-structured interview lasting approximately 5 min each. The interview protocol was refined by the authors year to year to better ascertain students’ experiences and perceptions (see Table 2 for the latest interview protocol). The interviews were conducted during the 2015, 2016, and 2017 summer informal STEM learning experiences. Over 40% of students were interviewed to gain an understanding of students’ perceptions of STEM, what they enjoyed most about the STEM learning experience, what was most challenging, and how the informal learning experience will help them in their STEM classes in a formal school setting. The interviews were audio recorded. The interviewer also took notes to conduct member checks during and at the end of the interview.

**Table 2:** Student participant interview protocol

Number	Question
1	What does STEM mean to you?
2	What is your favorite subject in school? Why?
3	What do you want to be when you grow up? Why?
4	What have you enjoyed about STEM Camp so far?
5	In what ways is STEM Camp preparing you for your math classes?
6	In what ways is STEM Camp preparing you for your science classes?
7	In what ways is STEM Camp preparing you for school in general?

**Fig. 1:**
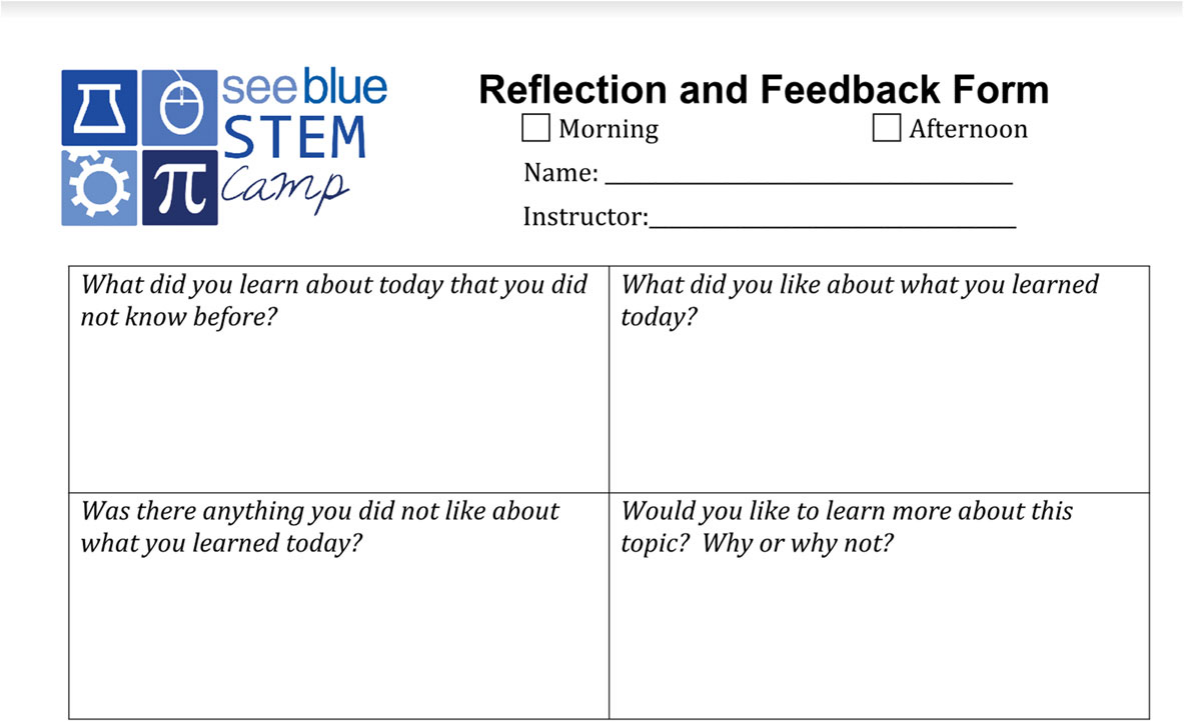
Daily reflection and feedback form the students completed after each session

In addition, the student participants completed a session reflection form (Fig. 1) at the end of each STEM content session (i.e., once a day). The STEM content session reflection was a handwritten by the students and were given to the students during the 2012, 2013, 2014, 2015, 2016, and 2017 summer informal STEM learning experiences. The purpose of the form was to collect students’ perceptions of the STEM content session, what the students learned, and provide feedback to the presenters. This data collection process occurred across all three sites. The final data set for this paper consisted of 320 qualitative artifacts. Of the 320 artifacts, 254 were unique interview transcripts from students across all 3 sites, with the majority (85%) coming from the founding site. Seventy-eight percent (197 of 254) of the students interviewed were from underrepresented populations in STEM. The remaining artifacts (66) were session reflections from across all 3 sites, with the majority (85%) coming from the founding site.

### Data analysis

All data were transcribed and a pseudonym was assigned to each participant. In order to create a reflection artifact, we took the transcribed session reflections from each unique participant in a content session offered in the summer informal learning experience and created one document with all participant reflections contained within it. For example, for the engineering design session at one university, all session reflections for that content session were combined together into one document to create a rich artifact that would help the authors draw out the “lived experience” of the students during that particular session.

We used an inductive approach to analyze the data, which incorporated systematic methods of managing data through reduction, organization, and connection (Dey 1993; LeCompte 2000). One member of the research team used initial coding to develop an early code list (Saldaña 2016). The initial coding primarily employed descriptive coding, “summarizing in a word or short phrase… the basic topic of a passage of qualitative data” (Saldaña 2016, p. 102). During this first cycle coding process, the descriptions began to paint a picture of the students’ most salient perceptions related to their participation in the summer informal STEM learning experience.

We, then, used the preliminary codes to establish further codes, which were used to code an initial set of interview transcripts and reflections. The entire author team then met to review the list of codes and revise the codes as necessary. All disagreements were discussed until a consensus was reached. Once a consensus was reached on the codes, a subset of the researchers (*n* = 4) coded the interviews and reflections using Dedoose (Dedoose Version 8.0.35 2018). Inter rater and intra rater reliability standards were set at 90% agreement. All four researchers exceeded the threshold of 90% agreement on both intra rater (ranged from 91 to 94%) and inter rater reliability (94.3%) which exceeded the minimum threshold of 90% needed for reliability analyses (James et al. 1993).

After the data were coded, four of the researchers conducted second cycle coding by pattern coding to appropriately group and label similarly coded data as a way to attribute meaning (Saldaña 2016). Pattern coding helped the researchers identify common themes, as well as divergent cases, looking across categories to see if there are underlying patterns to the responses (Delamont 1992). Once the initial themes were drafted, the entire research team reviewed the themes and supporting data to add clarity and content validity to the themes. During this review process, important questions were raised about the appropriateness of the themes and whether they were well supported. This process resulted in further identification of metathemes. All discrepancies were resolved during the final development of the overall themes.

## Results and discussion

As we used a situative lens to examine the research question of how participation in an informal learning environment influences students’ perceptions of STEM learning, three prominent themes emerged from the data. The informal learning environment (a) provided context and purpose to formal learning, (b) provided students opportunity and access, and (c) extended STEM content learning and student engagement.

### Context and purpose to formal learning

During the STEM summer learning experience, students programmed Lego robotics and completed several challenges. They were able to witness the applicability of STEM content. Jude explained, “I learned how to program and make robots, and I also learned how to use science and technology and math and engineering to build them” (Interview 2017). The students were at ease learning the STEM disciplines because they knew they were not learning the content in isolation. For example, Janae stated, “I learned a lot about mathematics. Like, the robotics. There’s a lot of logic in it, you know.” (Interview 2016). Luis further elaborated he had to “calculate how far it [the robot] is from the wall. And how far from the object… It [taught] me how to measure things more like without really thinking about it that much” (Interview 2016). The students recognized the importance of the STEM disciplines, and applied those skills to accomplish specific tasks during the robotics sessions.

Not only were the students able to apply STEM content to solve problems, they were able to see how what they were learning during the STEM summer learning experience was preparing them to be successful in the formal school setting. For example Kya explained,

I’m really interested in science and math, and so this place really helped me get ready for this year, this coming school year. I learned [many] things about science than I thought I would because almost every material is a polymer. I saw what smoking can do to your lungs and that is going to help me with health this year. Because my health teacher she like talks about how to stay healthy, what not to do, we have a conversation about drugs in the middle of the year. And so this is really going to help me. (Interview 2017)

Kya realized she would be able to take the knowledge she gained from camp and use it in her science and health classes. Similarly, James argued that one cannot do engineering without mathematics.

Engineering also focuses on math–like how if you measure a plane, if you measure the length or width of a plane, it shows… like the length and width, like base times height, length times width, stuff like that. And then you can do the math. Cuz [sic] in order to build a plane [you have] to do math, so it shows you different ways to do math problems while doing fun things. (Interview 2015)

It is important to note that students expressed they did not understand why they had to learn mathematics or science in school. To them, these subjects were disconnected from the real world, and they had to take the classes because that was what they were told they had to do in school. However, Erin articulated the importance of knowing the applicability of the discipline.

It’s helping me, and like showing me when I will need to use that math in real life problems, and it’s like helping me like understand why we need to learn math because I don’t like math very much. (Interview 2016)

The STEM summer learning experience provided a reason for students, like Erin, to learn subjects like mathematics in school, particularly for students who do not necessarily like the subject. Leslie agreed with Erin on how the camp provided meaning to the mathematics they were learning in school.

It's incorporating some of the math we've already learned into like STEM.It's giving us different ways to like apply the math that we will learn. So that we know why different equations or whatever what they’re for engineering would use some of the stuff like how to apply it into everyday life. So, it kind of gives meaning behind it. (Interview 2015)

The applicability of the activities completed during the STEM summer learning experience not only provided more context for the subjects students have in school, it also gave credence to help students understand why they learn the subjects in school. Luis commented the STEM summer learning experience helps “me in math classes because it gives me different scenarios to work with, and it helps me look at the problem in different ways not just in the same ordinary way” (Interview 2016). David realized mathematics involves more than understanding the basics. He suggested, “math isn’t all about like just 1 plus 1 stuff. It also involves calculating lots of things, not just equations” (Interview 2016). Shelby summarized the sentiments of many students in the camp,

I guess I feel like it’s giving us new ways to understand and see things, and it’s giving us things that we haven’t learned about; we’re just kinda getting it into our brains so we’re more prepared for our science classes. And, I just feel like it’s preparing us for what what’s going to be ahead of us and giving us ways to see things. (Interview 2016)

The students’ perceptions of the activities helped them to not only understand the purpose of the content they were learning, but realize the connections to what they are learning during the STEM summer learning experience can help them excel in the subjects they learn in school. In other words, providing the same content in a new environment was a catalyst for a positive change in how the students perceived future STEM content.

### Opportunity and access

Students recognized access to STEM curriculum and materials was often limited because of funding and resources in public schools. Cristina pointed to the lack of technology in her small, rural school as a major barrier to accessing STEM content. “I don’t have a lot of robotics in my school or computer things, and so I don’t learn a lot about these topics” (Interview 2017). Other schools offered STEM content such as robotics as an elective or after school club. However, this prohibited some students with working parents because “the schedule would be weird,” (Shelby Interview 2016) even though the student may “just love programming” (Shelby Interview 2016). The STEM summer learning experience provided access to robotics and other activities often offered outside of the school day or during times when students may have to choose between fine arts, academic support services, and other electives.

Students also commented their access to rigorous core content was also lacking in the formal school setting. Several students commented they had limited exposure to STEM because STEM was not part of their curriculum, and many stated they have science class “only once a year so [they] don’t really do anything” (Luis Interview 2016). However, other students stated they did have STEM as a part of their school curriculum, but were yearning for challenging content and an opportunity to dive deeply into STEM learning. Hilda explained that in schools, a teacher may not be able to “go into super detail just so she can get everything done in the year” (Interview 2017). The students were excited about the opportunities and access the STEM summer learning experience provided because they were “learning things [they] didn’t know before” (Jude Interview 2017). They were “learn[ing] new stuff and visit[ing] new stuff that [they have] never been before” (Frances Interview 2017). Students remarked about “want[ing] to learn more. That’s why [they] came back” (Melsia Interview 2015). When provided the opportunity to access STEM, students were engrossed in the learning and eager to experience the activities. For example, accessing STEM content inspired students to think about potential applications in the real world. After engaging in a session discussing the development and use of virtual reality (VR), Anthony noted VR could help:

...teaching other students about other worlds. Like, such, like not other worlds, other places, area. Like under the ocean or in space. We could, we could really use that in classrooms and sometimes even at home too with your, with parents if they sort of like, kind of forget some, some useful information. We could help them by using the VR. And we could probably add triggers to the VR and hand motions to see your hand, like an avatar hand so that we could see, pick up, and build some of our things. (Interview 2017)

A STEM concept previously perceived as science fiction was now a learning tool that could be evaluated and improved upon. Beyond connecting to formal classroom learning, students were also making connections between their experiences during the STEM summer learning experience and the application of those experiences to their personal lives and the real world. Once students have access to “all this in action, and [they can] see how it applies to real life” (Tamara Interview 2016), it is transformative.

The STEM summer learning experience partners are from a range of professions providing students experiences that are both broad and rich. The pedagogical approach of the STEM summer learning experience balanced guided learning and student exploration. Instead of sessions where professionals imparted knowledge to students, they were engaged in “little activities that they have that help [students] learn easily” (Shaun Interview 2016). Students remarked this was an essential element to their rich learning experience. Students had access to deep experiences with robotics and coding in the mornings and “the afternoon sessions are different for [them] every day, with different professors” (Leslie Interview 2015). Their access was not just brief encounters with STEM professionals. Students were spending hours with professors, STEM career professionals, and college students engaging in their real work in an authentic setting. One of the most discussed experiences was a field trip to an alternative energy research center. Simone remarked, “I think that was pretty cool because we got to walk around and kind of engaging conversations and stuff with professionals. So… and I learned a lot too, so that was fun” (Interview 2016). For many students, work with STEM professionals humanizes and normalizes the individuals. Denise reflected,

I’ve learned a lot here over the past couple of days. What I’m probably going to take with me is how there’s different types of people, and we’ve kinda gone over the fact that most people outside of like engineering world they think that scientists and engineers are people that don’t really have friends or are kinda secluded. But I’ve kinda learned that it’s not like that at all. They’re just normal people who do normal things like everyone else. (Interview 2017)

Adri also stated, “It’s really fun and it’s cool to see like campus and like what some people do as their jobs. And to learn that you could do that too” (Interview 2017). Learning about professionals’ “job and about their life story and how they got to where they were at” (Michael Interview 2017) brings them down from the pedestal and onto an equal playing field. In other words, it makes the professional attainable to the students.

### STEM content learning and student engagement

Students had an opportunity to experience activities they never experienced such as programming robots, cutting pigs, and playing with flies. These experiences extended their STEM content knowledge and piqued their interest and engagement. Several students expressed they really enjoyed doing the hands-on activities because that is how they learn. Shalea articulated,

I mean at my school we don’t really have many hands-on activities. It’s more of visual and audio learning. Like we do a lot of tests; we do a lot of things like that. And we really don’t get to do hands-on experimenting, and I am a pretty big hands-on learner, so it is hard for me to stay focused. It is hard for me to learn as fast as other people because I am more of a hands-on person. So, when there is a hands-on activity, I am really happy because I get [to] learn. I get to see. I get to feel. I get to touch, and I like how STEM camp^1^ incorporates that in a fun and awesome way. When we dissected pigs that helped [me] learn about biology in a way where it wasn’t like in health class, where here is a diagram of the human body. Here is a textbook. Read it through. We are going to read it through, we are going to learn about it. No, in this one we learned it in a fun and interesting way. We played bingo with pig parts. (Interview 2017).

Frankie stated,

This is like a summer school but way more fun. First of all, you have two snacks in a day and I usually have to wait a while for snack, and you get to learn about programming and not just boring writing in the boring workbook. I like the more hands-on. It really helped me I think. (Interview 2015)

More importantly, the students were excited that “we actually get to do things like robotics, and we get to like build. We got to learn the process of how like doctors take our DNA” (Adri Interview 2017). Jesse also commented, “I like building things. I mean like it’s fun. Like you get to do things with your hands. You get to move things. You get to like make your imagination things come true sometimes” (Interview 2017). The hands-on nature of the camp also allowed students to not just see how the content is used, but to practice doing the content. Paige described the process of DNA extraction:

Um, we took Gatorade, and mixed it with salt. So, it was like a Gatorade salt solution, and we put it in our mouth so it could absorb some DNA from like our cheeks without bursting it. And we take that and we put it in some detergent, and then we added some clear liquid. I think it was some sort of rubbing alcohol I don’t know. Then you could see like that parts of your DNA like build up. (Interview 2017)

Instead of simply learning about DNA, the students extracted DNA to explore biological concepts. Timmy reiterated, “I just like doing hands-on stuff and I love that STEM Camp lets us do like a bunch of things like hands-on activities and let us learn things and not just tell us what to do, but let us actually do them” (Interview 2016). The summer learning experience was “[m]ore actually doing things” (Jessica Interview 2016). For example, Sally said she liked “building the dam because we got to make up a lot of ideas and try to solve a problem with just the materials that we had” (Interview 2015). Lisa specifically mentioned the power of doing when she stated, “I actually [did] it myself. We didn’t have someone telling us what to do. Who gets to do that? And it helped me learn and that was really cool” (Interview 2017). The emphasis was on doing and seeing.

Students were not simply reading about STEM concepts or watching a video. Students’ learning about STEM was particularly peaked when they were able to interact with materials from STEM fields. Lab materials, software, and technology dominated conversations when students had access to supplies that were not readily available to them. Karena mentioned she “really enjoyed using the microscopes. Looking at larva. And being in the biology class in general. I loved looking at the organs” (Interview 2017). Learning about anatomy from diagrams, videos, and textbooks is not as rich of an experience for students as holding a human heart and brain in their hands.

Students commented on how the hands-on activities and experimenting made the content come to life. Josh, for example, emphasized, “I got to actually see a real brain, lung, organs, things like that, which I’ve never seen before, which was pretty interesting” (Interview 2016). Seeing organs was only part of the experience, though. Dolly described her experience dissecting pigs,

I know it sounds really weird, but at first it’s one of those things where you’re really nervous and like eh, ah, mmm, should I really do this. And it’s one of those things where your stomach, it’s like right before a rollercoaster like you’re stomach’s all tied up in knots and your brains like you want to like this. I’m asking you “do you want to do it, you can quit this right now, do you want to do this” but part of you is like “you know what I’m just gonna do this, I’m just gonna do this” and once you finally get to the top of that rollercoaster you’re like “hey, this is really really fun. I don’t want to stop.” And it’s like one of those things where it seems really gross and nerve-racking, but once you actually like start doing it you’re like “hey this is kinda fun in a disturbing way.” (Interview 2016)

The students were excited they had the opportunity to learn about biology and other science disciplines because many students commented, “We don’t do a lot of science at my school so it’s good to learn more about it” (Taylor Interview 2016). Stephan articulated they have to focus on specific standards, but “[STEM Camp] helps me with more background knowledge around all the subjects” (Interview 2017). The students understand that in the formal learning environment, their teachers may not have the time to go deeper into a subject. Hilda expressed,

I would say STEM camp, it kind of just, it kinda gives you a little bit of everything. Especially with, like, our science stuff. Our science teacher, she teaches us like, everything. She doesn’t go into super detail just so she can get everything done in the year…And this place, …the other half of the day you go into detail about every, like, little thing. Like today we were extracting our own DNA. And we’re talking about, like, the chromosomes and DNA and all this stuff inside of it. (Interview 2017)

Other students emphasized physical science concepts. For example, Jada explained, “I like the lessons. Like the lessons in the science lab because they were really fun cause we got to mix these chemicals together and see how they reacted to other chemicals and stuff. And it was really cool” (Interview 2017). The STEM camp experiences laid the groundwork for connections among disciplines and professions. While students “like to go into the lab and really, really experimenting with the lab coats and stuff,” (Suzanne Interview 2017), it is engaging in the STEM content in those environments that is so important to making it come to life.

[Students] were downstairs taking a tour of the engineering… [They] learned how the vibration and the echo and everything… how like if you talk nobody can hear you ‘cause the uh ectoplasm, something like that. Ectoplasm like, it’s on the walls and it’s pretty hard. So they have to use those little square things, I don’t really know what it’s called but it’s connected to the wall. And like you can like, it’s kinda loud in there but out there, you can’t even hear nothing. Like that’s awesome. I like doing that. (Melsia Interview 2015)

The students emphasized the active nature of participating in the content. Students saw real organs, dissected pigs, extracted DNA, mixed chemicals, made boats, and built levees. Students stressed learning through the activity. This same perspective was also evident as students’ engaged in robotics. Alyssa explained why she liked robotics:

Um I like how everything is really interactive and you learn stuff while having fun um because while you’re programming robots, you’re writing code and you’re programming, but it’s a lot of fun and you turn it into a competition and they make racetracks to make it more appealing to kids. (Interview 2016)

As the students completed the robotics challenges, they anticipated and looked forward to “going on to harder ones every time” (Becka Interview 2017). The students deepened their understanding of programming, and they did not give up when their robot did not do what was expected.

Like if you’re programming and you don’t do it right you can go back and fix it. So, it’s like kind of like a trial and error, with fixing things, and like if I do something wrong, I can go back and try to fix it. I just think about, like how, what did I do wrong that I could’ve done better. If it’s turning too far, then we’ll bring down the rotations. And then, if it’s uhm going too short, then we’ll just bring the rotations up more. (Cory Interview 2017)

Jordan shared,

I liked um programming the robots and learning um a little bit in each subject. When I’ve done something wrong, I’d go back and I would make the number or something, cuz you have to make the numbers, I would make the number a little bit higher, and if that wasn’t right I would make it in the middle between those two. It would teach me right from wrong. (Interview 2015)

Students did not talk about learning how to program by reading about it or by listening to a lecture or by completing a worksheet. The students, instead, emphasized learning to program by doing the programming. When they were wrong, as the student above described, they tried again. It was a problem solving process to program the robot to do what they wanted.

Melanie described, “I mean it’s fun having to programming the robots, but it’s really challenging. Program the robots, they do the wrong thing, but then you correct them and their mistakes” (Interview 2016). Unfortunately, many students discussed how they did not experience these “fun activities” at their school. The students were excited about all of the experiments and learning.

I’m addicted [to robotics], basically. Like as soon as they say like 10 minutes [left], I’m like rushing to get things done because it’s so fun and it takes, it actually takes effort. Not like you know, breeze through it and kinda know everything–like sometimes that happens at school. (Tonia Interview 2016)

Students expressed the STEM summer learning experience allowed them to be creative and use their mathematical skills when working with the robots. In a broader perspective, access to the professionals, curriculum, content, and environments central to the STEM camp experience built students up where they had previously felt inadequate or poorly adept. Morgan added,

Well I'm not really good at math, but I think this morning we learned about like different things that use different shapes that you've already learned like the great pyramids….sometimes there are shapes that you can use. Sometimes you have to draw them out and can use them to make cubes, pyramids, and things like…some of my sisters told me—she told me math in 7th grade is really hard to understand if you don't understand shapes so like…maybe because some things we've learned - some things don't apply but some—they will actually apply to what we learned but some of them will. (Interview 2015)

For this student, understanding something she perceived as really hard was a victory and confidence booster. For other students, they feel more prepared for classes because it’s helping me and like showing me when I will need to use that math in real life problems, and that I will need to, and it’s like helping me like understand why we need to learn math; because I don’t like math very much. (Erin Interview 2016)

Dolly said the STEM summer learning experience is

...preparing me for some daring things I might do. Like, you gotta be brave, you’ve gotta be willing to like actually throw yourself out in the world saying “hey I’m just gonna do this” because if you’re doing like a science project or something…you have to be optimistic about work, you’ve gotta be outgoing and I think going here is making be braver to do that…we’re being able to interact with things that will make me like learn be able to learn more things. (Interview 2016)

Students were able to connect their new learning to their futures. Some students thought more short term, “If I learn more about this topic it will better prepare me for middle school” (Walter 2014 Math Modeling Reflection), while other students were connecting their experiences to their distant future, “I want to become a doctor when I grow up, and to do so I need to know a lot about anatomy. Dissecting animals really helps me learn more” (Heather 2016 Anatomy Reflection). Students gained STEM knowledge because they were given the opportunity to access and engage in STEM activities and persevere.

## Conclusion

The goal of the STEM summer learning experience was to (1) provide upper-level elementary and middle level students, particularly underrepresented populations, access to a variety of STEM fields and STEM professionals in their workplace environment through authentic, hands-on learning activities, and (2) increase students’ interest in a STEM career. While one of the goals centered on STEM careers, the benefits of participating in the STEM summer learning experience also extended to students’ perceptions of future STEM learning. This study highlighted how the STEM summer informal learning environment influenced students’ perceptions of STEM learning. Specifically, the STEM summer learning experience provided students with context and purpose for formal STEM content. The use of project/problem-based learning allowed students to connect to real-world issues (STEM Task Force Report, 2014), such as seeing how mathematics is needed in the design and construction of planes, in the programming of robots, and in the calculations students used in measuring distance for their robotics challenges. By using authentic STEM workplaces, the STEM summer learning experience fostered a learning environment that extended and deepened STEM content learning while providing opportunity and access to content, settings, and materials that most middle level students otherwise would not have access to. Denise’s comments epitomized how interacting with STEM professionals normalized and humanized them. It allowed her to connect to the STEM community as a place where she can participate, practice, and belong (O'Connell et al. 2017).

Students also acknowledged the access they received to hands-on activities in authentic STEM settings and the opportunities they received to interact with STEM professionals were important components of the summer informal learning experience. In an era of budget cuts and pressure to cover material that will appear on standardized tests, schools are often limited in the access they can provide to in-depth content and authentic settings. Unfortunately, this contributes to the “receivement gap” (Chambers 2009, p. 418) that many students, particularly Black and Latinx students, experience. While policymakers focus extensively on outputs, such as achievement scores, less attention is focused on the inputs in and structures of education. The result is a system that does not provide equitable access or opportunity to authentic, engaging learning experiences that bring the content to life. As the students’ own comments showed, their participation in the summer informal STEM learning experience addressed the limitations of formal schooling through the experiences provided (Bell et al. 2009; Meyers et al. 2013). Thus, in the current system, one implication of this study is the importance of high-quality informal STEM learning experiences, such as those provided by the See Blue See STEM model, to increase students’ access and opportunity to engaging activities that contextualize and give purpose to their formal learning.

The findings of this research can also be considered to design authentic learning experiences in informal settings and to create purposeful contexts and settings in informal experiences. Providing access to meaningful contexts for learning (STEM Task Force Report 2014) and authentic settings is critical. While it is unrealistic to think every informal STEM learning environment would have access to scientists’ labs, creating partnerships with people in STEM careers is one way to provide a broader picture of what STEM is, where STEM happens, and who does STEM. This provides the meaningful exposure to a STEM community (O'Connell et al. 2017) and influences how students participate, practice, and belong in that STEM community.

While this study is important in highlighting the students’ perceptions of how participating in an informal STEM learning environment prepares them for future STEM learning, further research is needed to examine lasting impacts of participating in this type of informal learning. Exploring students’ future course taking patterns, success and perseverance in STEM-related courses, and choice of college majors and/or career are all areas needing further research.

## Abbreviations

NGSS: Next-generation science standards
PCAST: Presidential Council of Advisors on Science and Technology
STEM: Science, technology, engineering, and mathematics
U.S.: United States
VR: Virtual reality

## Footnotes

1 NOTE: Informal summer learning experiences are colloquially known as camp.

## References

[CR1] After-School Alliance (2011). STEM learning in afterschool: an analysis of impact and outcomes.

[CR2] After-School Alliance (2015). Full STEM ahead: afterschool programs step up as key partners in STEM education.

[CR3] Alexander KL, Entwisle DR, Olson LS (2007). Lasting consequences of the summer learning gap. American Sociological Review.

[CR4] Allsopp DH, Kyger MM, Lovin LH (2007). Teaching mathematics meaningfully: solutions for reaching struggling learners.

[CR5] Baran E, Bilici SC, Mesutoglu C (2016). Moving STEM beyond schools: students’ perceptions about an out-of-school STEM education program. International Journal of Education in Mathematics, Science and Technology.

[CR6] Barker BS, Larson K, Krehbiel M (2014). Bridging formal and informal learning environments. Journal of Extension.

[CR8] Bell, P., Lewenstein, B., Shouse, A. W., & Feder, M. A. (Eds.). (2009). *Learning science in informal environments: people, places, and pursuits*. National Academies Press. 10.17226/12190.

[CR9] Boekaerts M (1997). Self-regulated learning: a new concept embraced by researchers, policy makers, educators, teachers, and students. Learning and Instruction.

[CR10] Brown JS, Collins A, Duguid P (1989). Situated cognition and the culture of learning. Educational researcher.

[CR11] Brown PL, Concannon JP, Marx D, Donaldson CW, Black A (2016). An examination of middle school students' STEM self-efficacy with relation to interest and perceptions of STEM. Journal of STEM Education: Innovations and Research.

[CR12] Chambers TV (2009). The “receivement gap”: school tracking policies and the fallacy of the “achievement gap.”. The Journal of Negro Education.

[CR14] Creswell JW (2014). Research design: quantitative, qualitative, and mixed method approaches.

[CR15] Crotty, M. (1998). *The foundations of social research: meaning and perspective in the research process*. London, Sage.

[CR16] Dedoose Version 8.0.35 (2018). Web application for managing, analyzing, and presenting qualitative and mixed method research data.

[CR17] Delamont S (1992). Fieldwork in educational settings: methods. Pitfalls and perspectives.

[CR18] Denson CD, Hailey C, Stallworth CA, Householder DL (2015). Benefits of informal learning environments: a focused examination of STEM-based program environments. Journal of STEM Education: Innovations and Research.

[CR19] Dey I (1993). Qualitative data analysis: A user-friendly guide for social scientists.

[CR20] Finio B (2018). *Follow the Flow*.

[CR21] Guzey SS, Moore TJ, Harwell M, Moreno M (2016). STEM integration in middle school life science: student learning and attitudes. Journal of Science Education and Technology.

[CR22] Honey M, Pearson G, Schweingruber A (2014). STEM integration in K-12 education: status, prospects, and an agenda for research.

[CR23] James L, Demaree R, Wolf G (1993). Rwg: an assessment of within-group interrater agreement. Journal of Applied Psychology.

[CR24] Johri A, Olds BM (2011). Situated engineering learning: bridging engineering education research and the learning sciences. Journal of Engineering Education.

[CR25] Kelley TR, Knowles JG (2016). A conceptual framework for integrated STEM education. International Journal of STEM Education.

[CR27] King NS (2017). When teachers get it right: voices of black girls’ informal STEM learning experiences. Journal of Multicultural Affairs.

[CR28] Kirshner, D., & Whitson, J. A. (Eds.). (1997). *Situated cognition: social, semiotic, and psychological perspectives*. Mahwah, Lawrence Erlbaum Associates, Inc.

[CR29] Kitchen JA, Sonnert G, Sadler PM (2018). The impact of college-and university-run high school summer programs on students’ end of high school STEM career aspirations. Science Education.

[CR30] Klanderman DB, Moore WM, Maxwell MS, Robbert SK (2013). Creating problems and their solutions: service-learning through trinity mathematics triathlons, math nights, and math centers. PRIMUS.

[CR31] Kong X, Dabney KP, Tai RH (2014). The association between science summer camps and career interest in science and engineering. International Journal of Science Education, Part B.

[CR32] Langdon D, McKittrick G, Beede D, Khan B, Doms M (2011). STEM: good jobs now and for the future (Report #03–11).

[CR33] Larkins, D. B., Moore, J. C., Rubbo, L. J., & Covington, L. R. (2013). Application of the cognitive apprenticeship framework to a middle school robotics camp. In ) (Ed.), *Proceeding of the 44th ACM technical symposium on Computer science education* (p. 89–94). ACM). 10.1145/2445196.2445226.

[CR35] Lave J (1991). Situating learning in communities of practice. Perspectives on socially shared cognition.

[CR36] Lave, J., & Wenger, E. (1991). *Situated learning: legitimate peripheral participation*. Cambridge university press.

[CR37] LeCompte M (2000). Analyzing qualitative data. Theory Into Practice.

[CR38] NGSS Lead States (2013). Next generation science standards: for states, by states.

[CR39] Lincoln E, Guba I (1985). Naturalistic inquiry.

[CR40] Luehmann AL (2009). Students’ perspectives of a science enrichment programme: out-of-school inquiry as access. International Journal of Science Education.

[CR41] Martin LMW (2004). An emerging research framework for studying informal learning and schools. Science Education.

[CR42] Massey DD, Lewis J (2011). Learning from the “little guys”: what do middle and high school preservice teachers learn from tutoring elementary students?. Literacy Research and Instruction.

[CR43] McCombs, J. S., Augustine, C. H., & Schwartz, H. L. (2011). *Making summer count: how summer programs can boost children's learning*. Rand Corporation.

[CR44] Meredith CC (2010). Applied learning in teacher education: developing learning communities among pre-service candidates and urban elementary schools. The Journal of Human Resource and Adult Learning.

[CR45] Merriam SB (2009). Qualitative research: a guide to design and implementation (Revised and expanded from qualitative research and case study application in education).

[CR46] Meyers EM, Erickson I, Small RV (2013). Digital literacy and informal learning environments: an introduction. Learning, Media and Technology.

[CR47] Mohr-Schroeder, M. (2015*).* Track 3 panel session: national models for broadening participation. Invited panelist speaker at the 24^th^ National EPSCoR National Conference, Portsmouth, NH.

[CR48] Mohr-Schroeder MJ, Jackson C, Miller M, Walcott B, Little DL, Speler L, Schooler W, Schroeder DC (2014). Developing middle school students’ interests in STEM via summer learning experiences: see Blue STEM camp. School Science and Mathematics.

[CR49] Moote J, Williams J, Sproule J (2013). When students take control: Investigating the impact of the CREST inquiry-based learning program on self-regulated processes and related motivations in young science students. Journal of Cognitive Education and Psychology.

[CR52] National Council of Teachers of Mathematics (2000). Principles and standards for school mathematics.

[CR53] National Governors Association Center for Best Practices, Council of Chief State School Officers (2010). Common core state standards for mathematics.

[CR54] National Research Council (2009). Learning science in informal environments: people, places, and pursuits.

[CR55] National Research Council (2011). Successful K-12 STEM education: identifying effective approaches in science, technology, engineering, and mathematics.

[CR56] National Science Board (2016). Science and engineering indicators 2016 (Report No. NSB- 2016-1).

[CR58] National Science Foundation. (2017). Women, minorities, and persons with disabilities in science and engineering. Retrieved from https://www.nsf.gov/statistics/2017/nsf17310/static/downloads/nsf17310-digest.pdf.

[CR59] O'Connell KB, Keys B, Storksdieck M (2017). Taking Stock of Oregon STEM Hubs: accomplishments and challenges.

[CR60] Popovic G, Lederman JS (2015). Implications of informal education experiences for mathematics teachers’ ability to make Connections beyond the formal classroom. School Science and Mathematics.

[CR62] Saldaña, J. (2016). *Ethnotheatre: research from page to stage*. Routledge.

[CR63] STEM Task Force Report (2014). Innovate: a blueprint for science, technology, engineering, and mathematics in California public education.

[CR64] Sullivan FR (2008). Robotics and science literacy: thinking skills, science process skills and systems understanding. Journal of Research in Science Teaching.

[CR65] The Committee on STEM Education National Science and Technology Council (2013). Federal Science, Technology, Engineering, and Mathematics (STEM) Education: 5-Year Strategic Plan.

[CR66] The President’s Council of Advisors on Science and Technology (PCAST). (2010). *Prepare and inspire: K-12 education in Science, Technology, Engineering, and Math (STEM) for America’s future*. Executive Office of the President of the U.S.A. Retrieved from https://nsf.gov/attachments/117803/public/2a%2D%2DPrepare_and_Inspire%2D%2DPCAST.pdf

[CR67] Turner JC, Patrick H (2004). Motivational influences on student participation in classroom learning activities. Teachers College Record.

[CR68] U.S. Bureau of Labor Statistics. (2018). Occupational outlook handbook: fastest growing occupations. Retrieved from https://www.bls.gov/ooh/fastest-growing.htm.

[CR69] Van Manen, M. (1990). *Researching lived experiences*. Albany, State University of New York Press.

[CR70] Weinberg AE, Basile CG, Albright L (2011). The effect of an experiential learning program on middle school students’ motivation toward mathematics and science. RMLE Online.

